# Identification and Validation of an Individualized EMT-Related Prognostic Risk Score Formula in Gastric Adenocarcinoma Patients

**DOI:** 10.1155/2020/7082408

**Published:** 2020-03-20

**Authors:** Deyu Zhang, Siran Zhou, Bingrong Liu

**Affiliations:** Department of Gastroenterology, The First Affiliated Hospital of Zhengzhou University, Zhengzhou, Henan, China

## Abstract

**Background:**

The epithelial-mesenchymal transition (EMT) is a pivotal process for fibrotic disease, embryonic development, and wound healing. Moreover, some evidence has proven that the disorder of EMT also plays an important role in carcinogenesis, especially invasion and metastasis of various tumors (Ritchie et al., 2015). Additionally, gastric adenocarcinoma (GAC) is a common gastrointestinal malignancy which is the fourth most commonly diagnosed tumor. Our study is aimed at identifying the prognostic value of EMT-related genes in gastric adenocarcinoma.

**Methods:**

Firstly, high-throughput and clinical data were downloaded from The Cancer Genome Atlas (TCGA) database. 99 differentially expressed EMT-related genes (ERGs) were obtained in these gastric adenocarcinoma data. Secondly, GO and KEGG enrichment analyses show that EMT may promote gastric carcinogenesis. Next, 10 ERGs associated with prognosis of gastric adenocarcinoma patients are screened out by univariate Cox regression, and 6 pivotal prognostic ERGs (MMP8, MMP11, TFDP3, MYB, F2, and CNTN1) are identified through multivariate Cox regression. These 6 genes are confirmed with significant prognostic value in gastric adenocarcinoma through overall survival (OS) analysis. Finally, a risk score formula is constructed and tested in another gastric adenocarcinoma cohort from GEO.

**Results:**

99 differentially expressed EMT-related genes (ERGs) and their enriched pathways are identified. 10 ERGs are strongly related to the prognosis of GAC patients. A risk score formula of 6 prognosis-related ERGs used to predict the prognosis of gastric adenocarcinoma patients is identified and tested (risk score = 0.448115∗expression value of MMP8 + 0.378892∗expression value of MMP11 − 0.3226∗expression value of MYB + 1.322812∗expression value of TFDP3 + 0.325063∗expression value of F2 + 0.334197∗expression value of CNTN1).

**Conclusion:**

This study provides a potential prognostic signature for predicting prognosis of gastric adenocarcinoma patients and molecular insights of EMT in gastric adenocarcinoma, and the formula focusing on the prognosis of gastric adenocarcinoma can be effective.

## 1. Introduction

Gastric adenocarcinoma (GAC) is a common gastrointestinal malignancy which is the fourth most commonly diagnosed tumor and the second pivotal cause of cancer-related death toll worldwide [1]. The morbidity of GAC exceeds 400,000 cases, accounting for more than 40% of cancer rates worldwide, and the death toll has reached as high as 300,000 [2]. Over the past 20 years, there has been great progress in the diagnosis and treatment of GAC. However, the detection rate for GAC patients in the early stage remains poor, and most GAC patients are already in the progressive stage when diagnosed. Additionally, the prognosis of GAC patients in advanced stage remains poor, with a 5-year survival rate of less than 30% because of the high recurrence rate after surgery and chemotherapy resistance [3]. Hence, understanding the interaction mechanisms of critical molecules behind GAC occurrence and progression is significant, which will make great aid in the precaution of GAC and in the identification of novel effective therapeutic targets.

The epithelial-mesenchymal transition (EMT) is a pivotal process for fibrotic disease, embryonic development, and wound healing [4, 5]. Moreover, some evidence has proven that the disorder of EMT also plays an important role in carcinogenesis, especially invasion, and metastasis of various tumors, including gastric cancer [6–8]. Even though the profound mechanism that EMT acts in carcinogenesis has been studied a lot, the prognostic value of EMT-related genes and the biological function of these prognosis-related genes in EMT process remain rudimentary and inconclusive. To sum up, studies on the correlations and underlying biological process between EMT and gastric tumorigenesis should be valuable to develop some new ideas for cancer diagnostic, prognostic, and treatment.

In our present study, the correlation between EMT-related gene (ERG) expression and clinical data in 375 gastric adenocarcinoma (GAC) patients downloaded from The Cancer Genome Atlas (TCGA) was identified, and risk score (RS) was calculated as an independent index for overall survival (OS) prognosis based on ERGs. These findings indicate some pivotal EMT-related genes in gastric adenocarcinoma and can guide the following research on EMT of cancer. Furthermore, these research results could also raise an effective formula of risk score that could monitor EMT and predict prognosis in gastric adenocarcinoma patients.

## 2. Materials and Methods

### 2.1. Data Acquisition

The EMT-related gene (ERGs) list was downloaded from epithelial-mesenchymal transition gene database (http://dbemt.bioinfo-minzhao.org/download.cgi) which was used to collect human EMT-related gene. High-throughput sequencing mRNA expression data of ERGs and corresponding clinical data of Stomach Adenocarcinoma (STAD) cohort were obtain from the UCSC Xena datasets (https://xenabrowser.net/datapages/).

### 2.2. Differentially Expressed ERGs and Enrichment Analysis

The differentially expressed EMT-related genes (ERGs) in mRNA expression data of STAD cohort were identified by the limma package in R software (FDR < 0.05, ∣logFC∣ > 1) [9]. Visualization (with volcano plots and heatmaps) was executed with the ggrepel, ggplot, and pheatmap packages in R software. Biological processes (BP), cellular components (CC), and biological pathways in which these ERGs were significantly enriched were identified by gene ontology (GO) enrichment and Kyoto Encyclopedia of Genes and Genomes (KEGG) pathway analyses and visualized with the goplot package.

### 2.3. Establishing an Individualized Prognostic Index according to ERGs

After combining clinical data and mRNA expression data of ERGs, univariate Cox regression analyses were used to screen the differentially expressed ERGs with remarkable prognostic value. Then, multivariate Cox regression analysis was executed in these screened genes to filter out an independent prognostic predictor in ERGs. The clinical feature was identified, and the survival plot of these ERGs were drawn based on STAD-TCGA cohort using log-rank test. At last, the risk score (RS) for these prognostic ERGs was developed. The formula based on multiplied Cox regression coefficients can obtain the relative weight of genes in multiple Cox analysis. The clinical data of GAC patients were separated into high- and low-risk groups by the best RS value as the risk cutoff value. The survival curves were plotted by Kaplan–Meier (KM) method, and differences in the survival rates between high- and low-risk groups were assessed using log-rank test. Finally, these findings are tested in another GAC cohort in GEO datasets through survival analysis and ROC curve analysis.

### 2.4. Statistical Analysis

All statistical analyses and plots were implemented through R software 3.6.1 (https://www.r-project.org/). In detail, the relations between the mRNA expression of each ERGs and corresponding clinical data were analyzed through univariate Cox regression and independent prognostic factor, and their risk score (RS) was identified through multivariate Cox proportional hazards regression. The diagnostic and prognostic value of RS was calculated through receiver operating characteristic (ROC) curve in each dataset by “survival ROC” package in R software. Factors with *P* value < 0.05 was considered with statistical significance.

## 3. Result

### 3.1. Differentially Expressed ERGs

Hierarchical clustering of differentially expressed ERG expression levels.

RNA-seq and clinical follow-up data from TCGA-STAD datasets was downloaded, which contains 375 cancer samples and 32 normal samples.

**Figure 1:**
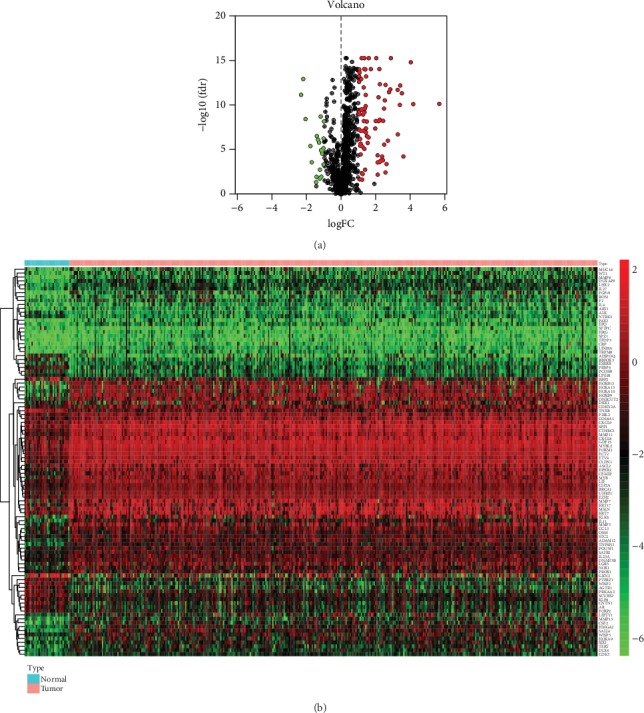
Differentially expressed EMT-related genes (ERGs) between gastric adenocarcinoma (GAC) and normal gastric tissues: (a) the volcano plot for the 1015 ERGs from TCGA-STAD cohort; (b) heatmap for screened ERGs.

1015 EMT-related genes (ERGs) are acquired from Human EMT Database. Then, the transcriptional expression of these 1015 ERGs in RNA-seq data was extracted and compared between normal samples and GAC samples through limma package in R software (logFC > 1, FDR < 0.05). The results indicated that there are 78 genes significantly upregulated and 21 genes significantly downregulated in GAC (Figures 1(a) and 1(b)). The specific logFC and FDR of differentially expressed genes are showed in Table 1.

### 3.2. Biological Functions and Significant Pathway Analysis Involved in the Expression of ERGs

**Figure 2:**
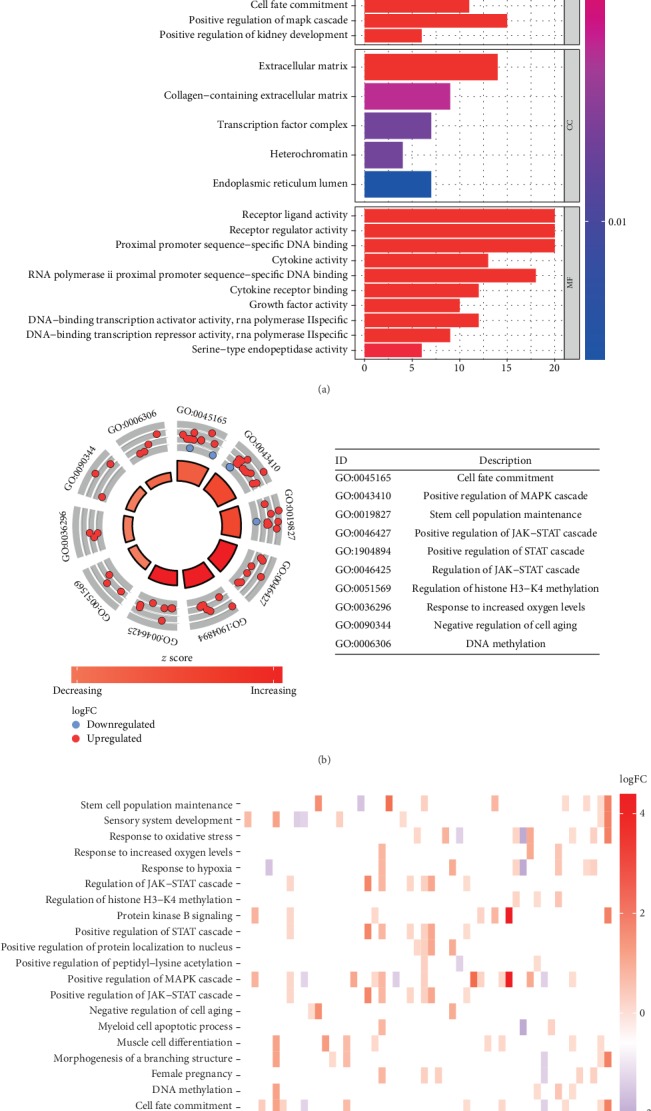
GO analysis of ERGs: (a) bar plot shows the enriched terms among ERGs (*P* < 0.005); (b) circle plot shows each term of the logFC of the assigned genes. (Red circles represent upregulated genes, and blue circles represent downregulated genes).

Biological functions and significant pathways analysis were executed of these 99 differentially expressed ERGs. Gene ontology (GO) enrichment terms are shown in Figure 2. The result of GO enrichment shows that the screened ERGs positively regulate the growth of GAC.

**Figure 3:**
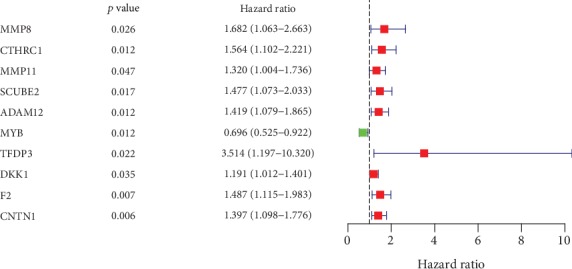
Univariate Cox regression analysis data of the prognosis-related ERGs in STAD-TCGA cohort.

**Figure 4:**
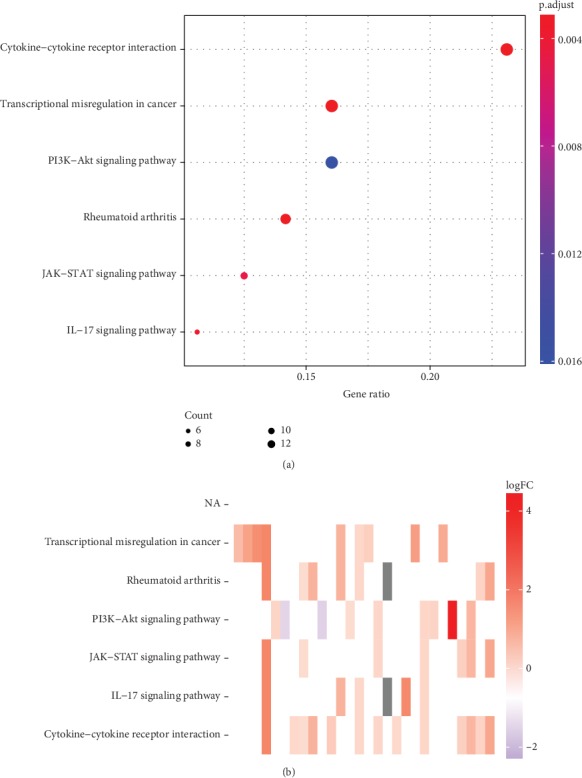
KEGG analysis of ERGs: (a) bubble plot shows the enriched pathways among ERGs (*P* < 0.005); red bubbles represent upregulation, and blue bubbles represent downregulation. (b) Heatmap shows each term of the logFC of the assigned genes.

KEGG pathway enrichment of these genes are shown in Figure 3. These results indicate that the differentially expressed ERGs can promote cancer-related pathways, including PI3k-Akt, IL-17, and JAK-STAT pathways, and may lead to transcriptional misregulation in cancer (Figure 4).

### 3.3. Identification of Prognostic ERGs

The correlations between the transcriptional expression of these 99 differentially expressed ERGs and clinical data were analyzed using univariate Cox regression (*P* value < 0.05 is considered a significant criterion). 10 genes were screened with significant prognostic value in GAC (Figure 3).

**Figure 5:**
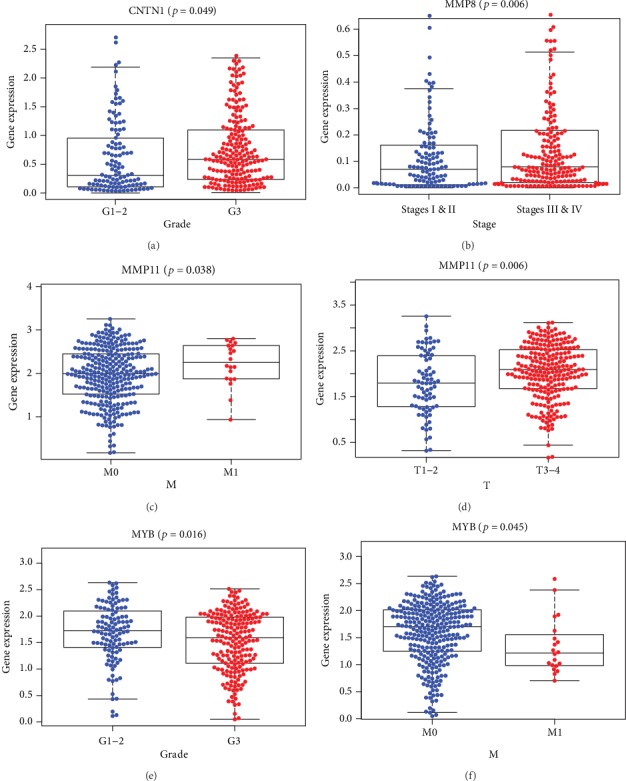
The relations between these genes are significant in multivariate Cox regression analysis and clinical index in STAD-TCGA cohort. Kaplan–Meier plots demonstrate the significant results (*P* < 0.05): (a) CNTN1 expression level and pathology grade, (b) MMP8 expression level and clinical stage, (c) MMP11 expression level and tumor metastasis, (d) MMP11 expression level and tumor size, (e) MYB expression level and pathology grade, and (f) MYB expression level and tumor metastasis.

**Figure 6:**
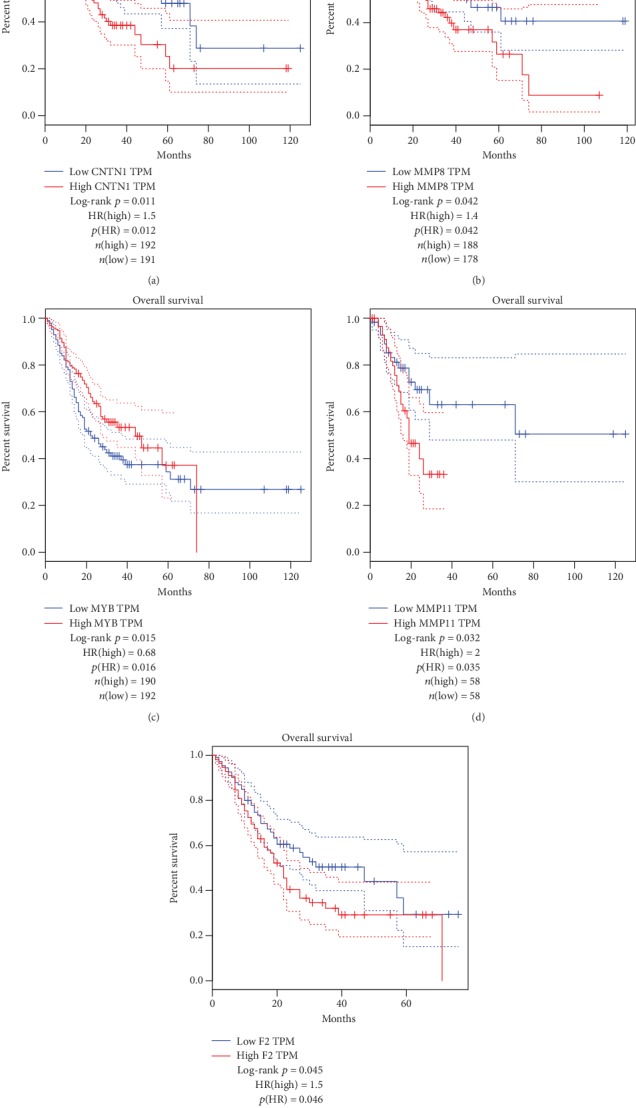
(a–e) Overall survival rates in Kaplan–Meier plots of each screened ERGs indicate that the changes of CNTN1, MMP8, MYB, MMP11, and F2 have considerable prognostic value in gastric adenocarcinoma (*P* < 0.05).

Then, multivariate Cox regression analysis of these 10 genes was implement, and MMP8, MMP11, MYB, TFDP3, F2, and CNTN1 are screened out which could be the independent prognostic predictor in GAC (*P* < 0.05) (Table 2). The relationships between these screened genes and clinical indexes are as follows (*P* < 0.05) (Figures 5(a)–5(f)). Moreover, 5 of these 6 genes, including CNTN1, MMP8, MMP11, MYB, and F2, are identified with significant prognostic value in gastric adenocarcinoma (Figures 6(a)–6(e)).

## 4. Construction and Definition of the RS

According to the result of multivariate Cox regression analysis as Table 1, the formula of the risk score (RS) is as follows: risk score = 0.448115∗expression value of MMP8 + 0.378892∗expression value of MMP11 − 0.3226∗expression value of MYB + 1.322812∗expression value of TFDP3 + 0.325063∗expression value of F2 + 0.334197 ∗ expression value of CNTN 1. In this formula, MYB has the only negative coefficient, indicating that high expression level of MYB is positively related with favorable prognosis while upregulated expression of MMP8, MMP11, TFDP3, F2, and CNTN1 is negatively related with the survival time of GAC patients.

**Figure 7:**
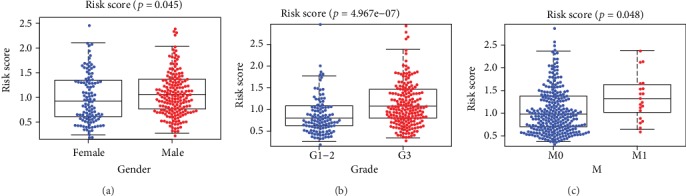
The clinicopathological significance of risk score in GAC from STAD-TCGA cohorts. RS value in different (a) patients' gender, (b) tumor pathological grades, and (c) pathological M stages.

Furthermore, we investigated the correlations for clinical index and risk score (RS) (Figures 7(a)–7(c)). The results of independent sample *t*-test analysis revealed that RS differentially expressed between male and female in GAC patients. Additionally, RS is also higher in patients with distant metastasis and pathological grades.

**Figure 8:**
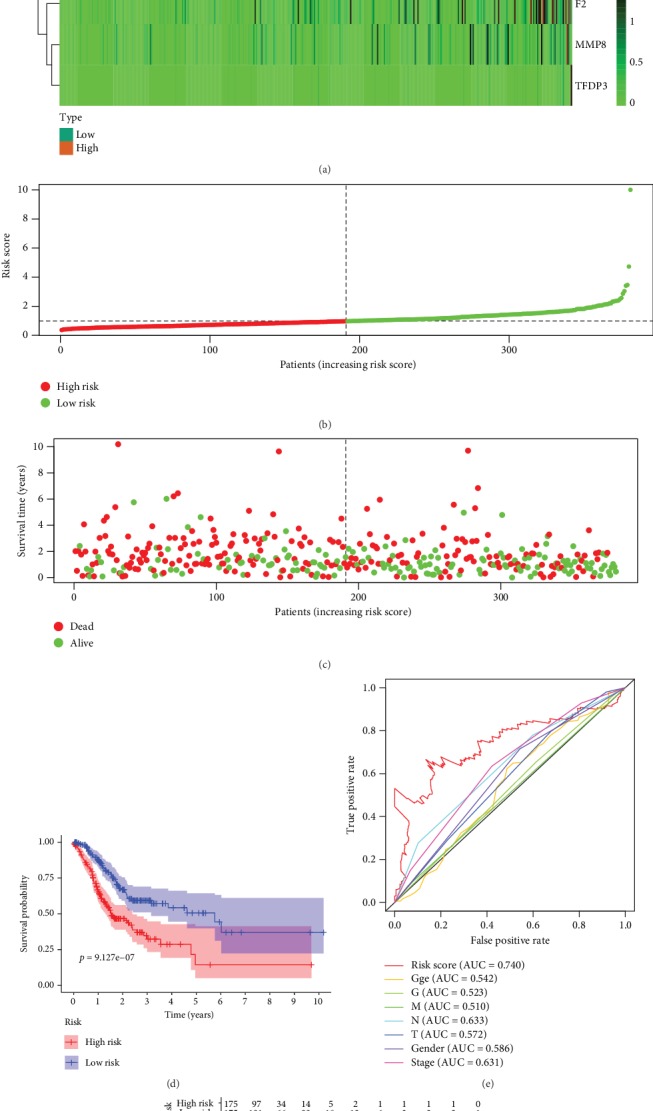
Risk score of GAC patients related to EMT. (a) The expression heatmap of these 4 pivotal ERGs in STAD-TCGA cohort. (b) The RS distribution of patients in STAD-TCGA cohort. (c) The number of patients in different risk groups. (d) Survival analysis reveals that the group of patients with high-risk score has significantly shorter overall survival time than that of the group with low-risk score patients. (e) ROC curve analysis of risk score with survival time in STAD-TCGA cohort. (f) The overall survival of patients divided into high and low-risk score in STAD-TCGA cohort.

To validate the pivotal role of RS in predicting the prognosis of GAC, survival analysis between the low-risk group and high-risk group divided by best cutoff was executed in STAD-TCGA cohort. Heatmap shows MMP11 and MYB are significantly downregulated while others are upregulated in samples from high RS patients (Figure 8(a)). The results of survival analysis reveal that GAC patients with high-risk score have significantly worse prognosis than GAC patients with low-risk score (*P* < 0.01; Figures 8(b)–8(d) and 8(f)). As visualized in Figure 8(e), ROC (receiver operating characteristic) curve analysis indicates that RS has considerable diagnostic and prognostic efficacy in GAC patients (AUC = 0.74).

**Figure 9:**
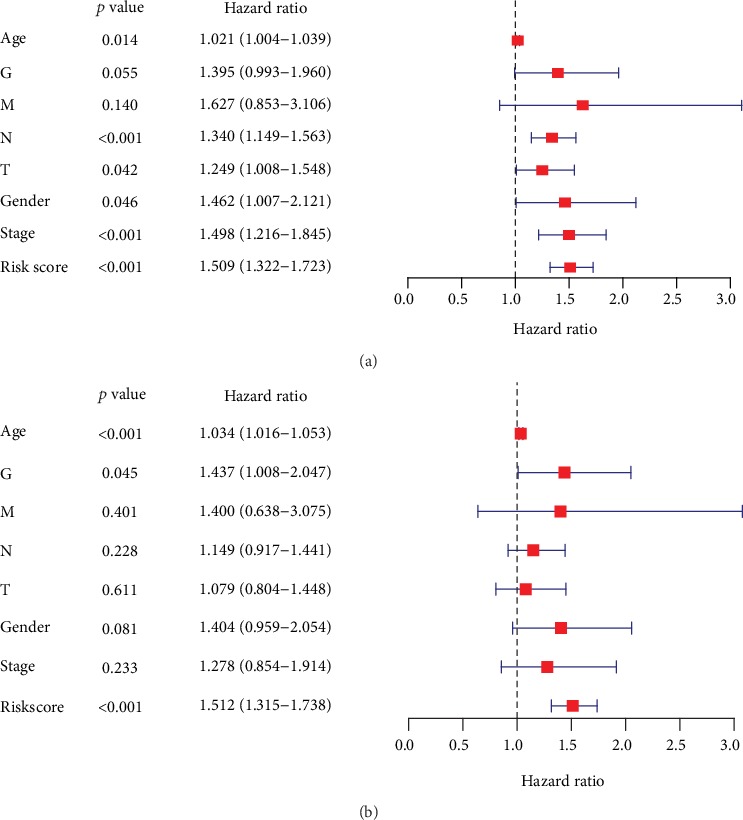
(a) Univariate Cox regression analysis of risk score (RS) in GAC cancer patients in TCGA datasets. (b) Multivariate Cox regression analysis of risk score (RS) in GAC cancer patients in TCGA datasets.

In addition, the reliability of RS in predicting the prognosis for GAC patients is further verified through univariate Cox analysis and multivariate Cox analyses in STAD-TCGA cohorts (Figures 9(a) and 9(b)). The results indicate that RS remained as an independent prognostic predictor in GAC patients in both univariate Cox regression analysis (HR = 1.509, 95%CI = 1.322‐1.723, *P* < 0.001) and multivariate Cox regression analysis (HR = 1.512, 95%CI = 1.315‐1.739, *P* < 0.001), adjusting with other clinical characteristic such as age, gender, tumor histological grade (grade), tumor pathologic stage (stage), and TNM staging (T, N, and M).

**Figure 10:**
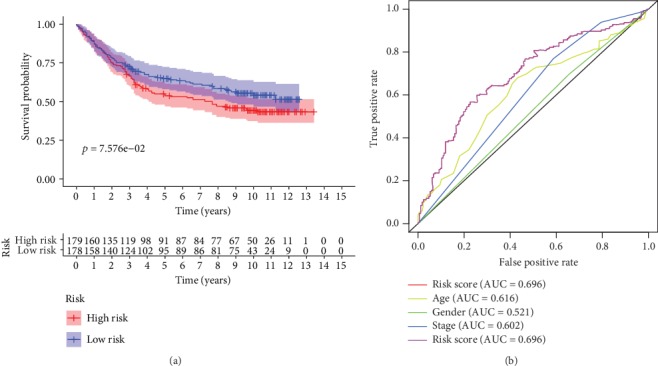
(a) Survival analysis about their RS in GSE84433 cohort. (b) Time-dependent ROC analysis of RS with survival time in GSE84433 cohort.

Finally, the formula and RS index above were tested in GSE84433 cohort from GEO datasets with 357 GAC tumor cases and corresponding follow-up information.  Figure 10(a) shows the significantly differential distribution of high-score group and low-score group through survival analysis (*P* < 0.01).  Figure 10(b) verified the prognostic and diagnostic value of RS (AUC = 0.696). The result indicated that RS can be a reliable predictor for predicting the prognosis of GAC patients.

## 5. Discussion

Gastric adenocarcinoma (GAC) is a major fatal type of cancer all around the world with a lack of reliable diagnostic and prognostic biomarker and therapeutic target [10]. It is of great importance for researchers to explore the new diagnostic and therapeutic strategy of GAC on these aspects. On the other hand, it has been widely proved that EMT is an important biological process in cancer, including gastric cancer. Specifically, EMT is characterized by downregulation of the epithelial cell properties and activation of the mesenchymal characteristic. Epithelial and endothelial cells are able to acquire a mesenchymal phenotype through EMT. The expression of certain genes, including those for cadherins, integrins, and many transcription factors, is reprogrammed in the EMT. EMT is thought to be one of the major mechanisms that determine invasion and metastasis of cancer cell [11]. However, most of the studies focus on the function and mechanism of some EMT-related genes and the diagnostic and prognostic value of overall EMT-related genes(ERGs) has not been widely elucidated in GAC.

In this study, we collected the high-throughput data about the transcriptional expression of 375 GAC samples and 32 nontumor sample with their corresponding clinical data from TCGA database. Among these, we screened out the differentially expressed ERGs between GAC samples and nontumor gastric samples. Then, the differentially expressed ERGs are listed and analyzed, and the pivotal functional clusters and pathways of the differential expressed ERGs are identified. Interestingly, major differentially expressed ERGs in the cluster associated with negative regulation of cell aging are upregulated, indicating that EMT process promotes the cellular immortalization of GAC. Another novel finding is DNA H3-K4 methylation, in which the MAPK pathway, JAK-STAT pathway, PI3K-Akt pathway, and IL-17 pathway are involved in EMT process of GAC. The enrichment analysis verifies the affinity between EMT and these enrichment pathways, which reveals some therapeutic target of GAC in EMT process and may lead further research about the internal mechanism. In previous studies, H3K4 methylation has been found widely existing in EMT of various cancers and our finding verified it through high-throughput data [12, 13]. Interestingly, there was no previous study that reported that the IL-17 pathway participates in EMT process while our study revealed that the IL-17 pathway may play an important role in EMT process. The researches about IL-17 pathways are needed in further practice. Univariate Cox regression analysis with survival data indicates that 10 ERGs were related to prognosis of GAC patients in STAD-TCGA cohort. Among these 10 genes, 6 genes, including MMP8, MMP11, MYB, TFDP3, F2, and CNTN1, are screened out as independent prognostic predictor, and a formula about risk score (RS) was raised through multivariate Cox regression analysis. MMP8 and MMP11 are two members of matrix metalloproteinases (MMPs), which are used to degrade extracellular matrix (ECM) components and deeply participated in EMT [14]. MYB is another critical transcriptional factor for EMT. Tao et al. reported that b-Myb regulates snail expression to promote EMT in breast cancer [15]. Qu et.al reported c-Myb promotes development of colorectal cancer through EMT [16]. Our study has revealed the significant role of MYB in GAC tumorigenesis, and further studies are needed to identify the related protein and protein interaction about MYB in GAC. TFDP3 is another MET-related gene which can also induce apoptosis and autophagy in breast cancer and prostate cancer [17–19]. F2 and CNTN1 has been reported to promote EMT in gastric cancer [20]. As for our present study, we found that upregulation of MMP8, MMP11, TFDP3, F2, and CNTN1 adds the risk score (RS), and overexpression of MYB could decreases RS. Additionally, upregulation of CNTN1 is significantly related with worse pathologic grade. Overexpression of MMP8 is significantly associated with higher clinical stage, overexpression of MMP11 is significantly associated with bigger tumor size, and low expression of MYB is significantly related to worse pathologic grade and metastasis. The validation gastric cancer cohort from GEO database shows basic consistence with our findings in STAD-TCGA cohort. To sum up, we speculate that MMP8, MMP11, TFDP3, F2, and CNTN1 can promote the progression of GAC while MYB could be a tumor suppressor in GAC.

So far, most of the cancer-related genes identified in bioinformatic methods are analyzed individually by single, which cannot be a comprehensive reflection of carcinogenesis process, and the ability of these single genes remains poor in diagnosis and prognostic prediction [21–24]. Moreover, most studies research on cancer of an organ without focusing one specific type of cancer or one specific process in carcinogenesis. In our point of view, it is more useful to identify a cluster of genes with clinical value in one specific cancer-related process, and this can be the highlight in the present study. However, this study also has some imperfection. Firstly, the study can be more valuable if further basic experiments are done on these 6 screened genes to explore their deep mechanism in EMT. Secondly, the validation group in our study is based on GEO database. A cohort based on PCR results from samples of clinical patients and corresponding follow-up can enhance the quality of our present study. The study on these flaws can be our next goal about precision therapy research in GAC.

In conclusion, our study indicates some related genes and pathways in the EMT process of GAC, and MMP8, MMP11, TFDP3, MYB, F2, and CNTN1 can be pivotal ERGs in GAC. The risk score formula can be a sensitive diagnostic and prognostic predictor in GAC. Risk score = 0.448115∗expression value of MMP8 + 0.378892∗expression value of MMP11 − 0.3226∗expression value of MYB + 1.322812∗expression value of TFDP3 + 0.325063∗expression value of F2 + 0.334197∗expression value of CNTN1.

## Figures and Tables

**Table 1:** Expression patterns of these 99 differentially expressed EMT-related genes (ERGs) in GAC samples and paired nontumor samples in STAD-TCGA cohorts.

Gene symbol	logFC	FDR	Gene symbol	logFC	FDR	Gene symbol	logFC	FDR
PEBP4	-2.33137	6.57E-12	CDK3	1.114647	7.39E-08	MMP11	2.017559	4.86E-16
WISP2	-2.20329	1.10E-13	CXCL9	1.142761	2.91E-10	LIN28A	2.094377	4.17E-05
ADIPOQ	-2.07249	3.55E-09	GDF15	1.15038	1.10E-13	EPO	2.095496	0.000266
GKN2	-1.79083	3.82E-06	ECT2	1.16946	4.86E-16	TFDP3	2.099731	0.006324
RBFOX3	-1.7153	0.000257	ART1	1.171375	0.004225	HOXB9	2.147629	1.24E-11
RBP2	-1.46036	0.044415	CXCL8	1.192881	2.91E-10	DLX4	2.160591	6.03E-09
ESRRB	-1.43626	0.011113	DKK1	1.202199	0.022856	CEMIP	2.197806	8.34E-15
CPEB1	-1.41884	2.85E-07	OSM	1.216572	4.38E-06	CRP	2.216016	0.000239
AGTR1	-1.35077	7.34E-07	IL4	1.228225	0.005411	MMP3	2.223194	4.14E-09
CNTN1	-1.28961	1.54E-06	MYB	1.254482	1.10E-09	TNFSF11	2.22423	4.18E-13
SFTPC	-1.24956	0.017655	MSLN	1.263453	7.45E-08	MUC16	2.266762	0.000175
PCDH9	-1.2381	0.00175	SPP1	1.290127	9.65E-13	HRG	2.356892	5.88E-05
SCUBE2	-1.19769	1.83E-09	CCL3	1.30075	5.84E-09	SPZ1	2.400927	0.000154
FOXP2	-1.17188	1.15E-05	UHRF1	1.325638	8.34E-15	TERT	2.413485	5.63E-09
ALK	-1.16027	0.01087	CDKN2A	1.331215	7.96E-06	HOXA9	2.467816	2.30E-10
AR	-1.10839	2.67E-05	CIP2A	1.332805	4.86E-16	TRPM8	2.476179	2.65E-06
PRKAA2	-1.09525	1.25E-05	SATB2	1.333976	6.11E-10	NOX1	2.510433	2.75E-11
NTRK3	-1.03347	6.74E-06	STC2	1.352507	2.31E-10	F2	2.550878	0.003578
KLF8	-1.02809	0.000461	WISP3	1.372624	0.001766	IL11	2.55524	5.02E-13
PTPRZ1	-1.02559	5.49E-07	LIF	1.386116	9.05E-15	LEFTY1	2.611598	0.000403
TNXB	-1.0026	6.46E-09	EPHB2	1.405853	1.22E-10	WT1	2.69005	9.60E-07
F2RL2	1.013227	5.33E-10	CLDN1	1.408831	6.87E-12	HOXA13	2.754086	3.25E-12
MYBL2	1.021142	2.30E-13	KRT17	1.419692	1.09E-07	HOXA10	2.828267	1.59E-12
EZH2	1.022635	5.28E-14	MMP7	1.423215	3.95E-08	ADAM12	2.861361	4.86E-16
COL8A1	1.029066	5.25E-08	ETV4	1.430788	5.58E-14	HMGA2	3.253388	1.82E-12
BRCA1	1.035784	9.05E-15	ONECUT2	1.468478	6.67E-09	ROS1	3.263434	1.86E-07
IL23A	1.037939	9.92E-10	KLK6	1.475237	2.90E-07	MMP13	3.382188	8.96E-11
FOXM1	1.053511	1.98E-14	IL27	1.521816	1.72E-06	DUXAP9	3.402413	5.84E-13
PROX1	1.057225	0.021408	LGR5	1.573145	4.08E-07	CSF2	3.500901	4.33E-12
POU5F1	1.08384	6.04E-07	CTHRC1	1.584522	4.86E-16	PAX2	3.586424	5.71E-05
ASCL2	1.090571	3.53E-06	DNMT3B	1.703268	8.34E-15	SALL4	4.014737	1.42E-15
KRT7	1.112453	2.04E-06	SIX1	1.957393	4.02E-08	MMP8	4.154083	7.24E-11
HOXB13	1.113473	3.37E-05	LHX2	1.986649	5.84E-09	FGF19	5.662702	6.87E-11

**Table 2:** Results of multivariate Cox regression analysis for ERGs in STAD-TCGA cohort.

Gene symbol	Coefficient	HR	HR.95L	HR.95H	*P* value
MMP8	0.448115	1.565359	0.968086	2.531126	0.047598
MMP11	0.378892	1.460665	1.097001	1.944887	0.009494
MYB	-0.3226	0.724261	0.521338	1.006167	0.044441
TFDP3	1.322812	3.753962	1.240255	11.36237	0.019231
F2	0.325063	1.384118	1.024723	1.869562	0.034075
CNTN1	0.334197	1.396818	1.059458	1.841604	0.017814

## Data Availability

The data used to support the findings of this study are included within the article. The gene expression data can be accessed on The Cancer Genome Atlas (TCGA) and Gene Expression Omnibus (GEO).

## Supplementary Materials

Supplementary Materials The data of risk score of GAC patients related to EMT (Figure 8).
